# Validation Assessment of a Pain Interference Questionnaire among Student Pharmacists

**DOI:** 10.3390/pharmacy9040170

**Published:** 2021-10-15

**Authors:** Megan Whaley, Nouf Bin Awad, Terri Warholak, David Rhys Axon

**Affiliations:** Department of Pharmacy Practice and Science, College of Pharmacy, University of Arizona, Tucson, AZ 85721, USA; swhaley@pharmacy.arizona.edu (M.W.); nbawad@pharmacy.arizona.edu (N.B.A.); warholak@pharmacy.arizona.edu (T.W.)

**Keywords:** pain interference, quality-of-life measurement, quality-of-life outcomes, patient-reported outcomes, validity studies, physical functioning, chronic pain, student pharmacists

## Abstract

Validation studies of pain interference instruments used among student pharmacists are rare yet essential for understanding their appropriate use and interpretation in pharmacy education and practice. This study conducted validation and reliability assessments of a five-item Pain Interference Scale previously administered to student pharmacists. Construct validity was assessed using Rasch analysis. Unidimensionality was measured using: point-biserial measure correlations; percent of raw variance explained by items; difference between expected; variance modeled by items; and Rasch model fit. To assess scale functioning, response frequency distribution, observed average and sample expected logit distribution, Andrich logit distribution, item separation, and item reliability were assessed. Visual examination of the Item-Person Map determined content validity. Items explained 64.2% of data raw variance. The difference between raw variance modeled and observed was 0.6. Point-biserial measure correlations were >0.77. Item mean-square infits were 0.7–1.3 while outfit measures were 0.72–1.16. There were >10 responses per response category, response frequency and Andrich thresholds progressively advanced, and observed average and sample expected logits advanced monotonically, Andrich logits = −2.33–1.69, item separation = 2.61, and item reliability = 0.87. Item probability curves indicated response categories were minimally yet adequately distinct. Cronbach’s alpha = 0.93. The Item-Person Map had a ceiling effect indicating content gaps. In conclusion, the pain interference instrument has acceptable construct validity yet contains content gaps. Additional difficult items should be added to the instrument to better capture pain interference among student pharmacists.

## 1. Introduction

The Department of Health and Human Services and the Institute of Medicine have made recent statements acknowledging chronic pain and its impact on health outcomes [1,2]. In 2019, the National Center for Health Statistics estimated that 20% of adults in the United States (US) were living with chronic pain and 7% were living with chronic pain that had significant pain interference [3]. Pain interference can be defined as the perceived extent to which pain obstructs an individual’s engagement in daily activities including recreational, emotional, physical, or cognitive activities [4]. Measuring pain interference has been recommended for use in clinical practice and clinical trials as a proxy for measuring physical functioning or extent of disability associated with pain [4,5,6,7].

Pain has a considerable economic cost. For example, the Institute of Medicine estimated the annual cost of pain in the US was USD 560–635 billion in 2008, with USD 99 billion of that cost captured by federal and state programs such as Medicare, Medicaid, Department of Veterans Affairs, workers compensation and others [1]. More recently, Zhao et al. estimated that the socio-economic burden of moderate to severe pain interference in daily activities compared to no or mild pain interference in the lives of US individuals living with osteoarthritis was USD 13.7 billion in annual all-payer medical expenses and USD 0.5 billion in wages lost [8]. Additionally, compared to adults with osteoarthritis and no or mild pain interference, the annual per-capita incremental all-payer medical expenses of adults with osteoarthritis and moderate to severe pain interference was USD 2849 (95% CI = USD 2791–2907) [8].

From a clinical and public health perspective, it is important to understand how pain manifests and interferes in individuals’ lives to direct tailored resources towards interventions that effectively improve the functionality of individuals with chronic pain [1,9]. The Centers for Disease Control and Prevention 2016 guidelines on treating chronic pain acknowledge the complex biopsychosocial nature of pain while advocating for multidisciplinary therapies, including nonpharmacological therapies such as exercise therapy and cognitive behavioral therapy [10]. Previous research has found that individuals with pain use several pain management strategies simultaneously, including both pharmacological and non-pharmacological strategies [11,12].

Pharmacists and student pharmacists are uniquely situated in the health care system to help patients achieve greater functionality in life. Because pharmacists’ clinical judgement is not only informed by their training but also by their life experience, it is of interest to understand their experiences with pain and strategies for pain management [13].

Pain interference, such as quality of sleep, depression, or satisfaction, is a subjective reality, and, therefore, is difficult to measure directly. This has resulted in a multitude of patient self-reported instruments that measure pain interference in different ways, including the 7-item Brief Pain Inventory, 56-item PROMIS^®^ Pain Interference (PROMIS-PI), and 2-item Short-Form 36 Bodily Pain Scale [5,14,15,16,17]. Some measures include questions which are disease specific while others may be generic enough as to be able to be applied across types and locations of pain. Therefore, it is essential to examine the validity and reliability of self-report instruments intending to measure pain to gauge the appropriateness of their implications in each population they purport to measure [18].

A recent exploratory study characterized the pain characteristics, management strategies, and outcomes of student pharmacists at the University of Arizona College of Pharmacy using a 24-item paper-based survey instrument administered in fall 2015 [13]. A full description of this study is available elsewhere [13]. This instrument was adapted from a study by Breivik et al. which was initially designed to assess the prevalence, treatment, and impact of chronic pain in 15 European Countries and Israel [19]. The 24-item instrument contained a five-item Pain Interference Scale which will be referred to throughout as the “Pain Interference Scale” [13]. Though the Breivik instrument was rigorously developed, it was designed to be administered to European citizens over the age of 30 [19], rather than a student population in the US. Therefore, there is a need to assess the validity and reliability of this five-item Pain Interference Scale for use with student pharmacists in the US. In particular, there is value in using Rasch analysis to obtain evidence of validity. Rasch analysis assesses the extent to which scaled items function to produce ordinal level data that approximates interval level data and behave like a Guttman Scale, a scale characterized by a positive correlation between the ability of respondents and the probability of respondents endorsing more difficult items [18].

The objective of the present study was to examine the validity and reliability evidence for the five-item Pain Interference Scale used in the Axon et al. study [13] using Classical Test (CTT) and Item Response Theory (IRT) methods.

## 2. Materials and Methods

### 2.1. Pain Interference Scale

The present study examined the five-item Pain Interference Scale from a pain management questionnaire administered to student pharmacists at a public, United States university [13]. The Pain Interference Scale had 172 complete responses and included five items that addressed pain interference in daily activities, ability to participate in leisure activities, ability to work, ability to attend school, and relationships with other people [13]. A five-point response scale was used with the following scoring method: ‘Not at all’, ‘A little bit’ = 1, ‘Somewhat’ = 2, ‘Quite a bit’ = 3 and ‘Very much’ = 4 [13]. For the purposes of this analysis and ease of interpretation, the response options were flipped so that higher scores would represent less pain interference (i.e., ‘Very much’ = 0, ‘Quite a bit’ = 1, ‘Somewhat’ = 2, ‘A little bit’ = 3, ‘Not at all’ = 4). Table 1 displays the original questionnaire structure.

### 2.2. Content Validity

Face validity was reviewed independently by two pharmacist researchers with experience in pain management. The researchers then came to consensus on whether each item appeared to measure an aspect of pain interference. This was an informal procedure involving their clinical judgement, experience, and pharmacy training. Because only two reviewers assessed face validity, it was not appropriate to calculate the Content Validity Index (CVI). Content validity is the extent to which the items address the breadth and depth of the phenomenon represented by the theory or domain [18]. To assess content validity, we visually examined the Rasch Item-Person Map to determine whether the distribution of items along the logit scale of difficulty approximately mirrored the distribution of respondent ability [20]. If there were gaps in content between items, they were assessed for significance using the standard error for the respective items and logit score in the z formula [20]. Any items which appeared at the same item difficulty were evaluated for content redundancy as defined by Wright and Masters [20].

### 2.3. Construct Validity

Construct Validity is the degree to which the items on an instrument address the same underlying conceptual dimension as it manifests in the studied population [18]. The dimensionality was assessed using the table of standardized residual variance in the Rasch Winsteps output [21]. Specifically, the following were evaluated: the raw variance of the sample explained by the measures; the difference between the raw variance expected by the model and that observed in the sample; the unexplained variance in the first contrast; and the point-biserial measure correlation. Ideally, 60% or greater of the sample’s variance will be explained by the measures (Rasch item difficulties, person abilities and rating scale structures) and the difference between the variance observed and that predicted by the model is approximately 0% [21]. Additionally, the acceptable threshold for the unexplained variance in the first contrast is an eigen value of less than 3 and point biserial correlations are greater than 0.2 [21]. If these thresholds are met, Linacre suggests that there may be sufficient evidence to claim that the items form a unidimensional construct [21]. Parameter thresholds for all tests are included in Table 1.

### 2.4. Scale Functioning

To assess scale functioning, we used criteria established by Linacre [22]. Specifically, we evaluated the rating scale categories to see if they met the following criteria: at least 10 observations per response category; regular advancement in response frequencies across response options; observed average and sample expected responses advanced monotonically with category; category infit and outfit mean-square (MNSQ) were less than 2 and optimally between 0.6 and 1.4; the anchors have the largest MNSQs; Andrich thresholds advanced with category by at least 1.4 logits but less than 5 logits; and coherence of ratings which imply measures and measures which imply ratings of at least 40% [22]. When the Andrich thresholds observed average and sample expected responses advance monotonically, it demonstrates that the scale functions as a Guttman Scale and the data fit the Rasch model [18,21]. In addition, we evaluated the response probability curves to ensure each response option has a distinct and well separated peak from other responses. Distinct and well separated peaks indicate that response options are specific enough to differentiate between persons of differing abilities. If peaks are not distinct then it is possible that respondents may be unable to distinguish between response options and it may be ideal to have fewer response options [20]. If the data fit the Rasch model, the scales’ ordinal level data may approximate interval level data and it may be appropriate to perform parametric Classical Test Theory analysis on the data [23].

### 2.5. Reliability

Internal consistency of the Pain Interference Scale was assessed using Cronbach’s alpha as well as the Rasch reliability and item separation indices. In this study we adopted the guidance on unacceptable (α < 0.6), acceptable (0.6 ≥ α > 0.9) and high (α ≥ 0.9) Cronbach’s alpha thresholds provided by the National Quality Forum [24]. An item separation of 2 implies an item reliability of 0.8 [25]. Item separation values greater than 2 and item reliability values greater than 0.8 indicate that the items represent distinct aspects of the domain [25]. Item fit to the Rasch model was assessed using criteria from Linacre for infit and outfit MNSQ and z-standard (ZSTD) values for each item [22]. A MNSQ in the range of 0.6 to 1.4 and ZSTD in the range −2 to 2 indicates that items fit the Rasch model well [22]. Parameter thresholds for all tests are included in Table 1. Rasch analysis was conducted using Winsteps 4.8.0. Cronbach’s alpha was calculated using SAS Software 9.4 (SAS Institute Inc., Cary, NC, USA).

## 3. Results

### 3.1. Content Validity

**Figure 1:**
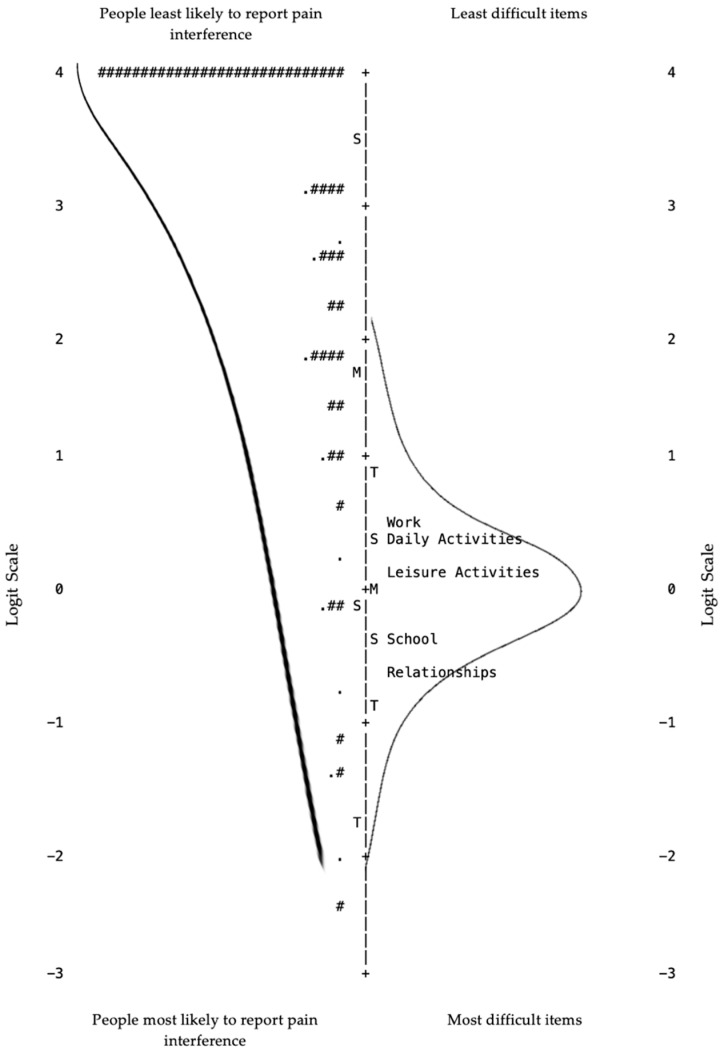
Item-Person Map. Key: “.” = 1 or 2 persons; “#” indicates 3 persons; “##” = six persons; “###” = nine persons; “####” = twelve persons; “#############################” = 87 persons; “M” = mean; “S” = standard deviation; “T” = two standard deviations; “Work” = pain interferes with ability to work; “Daily activities” = pain interferes with daily activities; “Leisure Activities” = pain interferes with ability to participate in leisure activities; “School” = pain interferes with ability to attend school; “Relationships” = pain interferes with relationships with other people.

Both pharmacist researchers with experience in pain management confirmed that the items appeared to measure pain interference. Figure 1 details the Item-Person Map. To examine the Item-Person Map, the likelihood of the person responding that pain interferes with their life as well as the difficulty of the item are measured in logits on the vertical axis. Persons least likely to report that pain interfered in their lives and the least difficult items to endorse that pain interfered in that area of life are at the top of the axis. Persons most likely to report that pain interfered with their lives and most difficult items to endorse are at the bottom. Ideally for optimal scale functioning, the mean and distribution of respondent ability will roughly mirror the mean and distribution of item difficulty on either side of the vertical axis. Essentially this would indicate that the items were appropriate considering the ability of the respondents. Alternatively, if the distribution curves are offset from one another, e.g., the mean of the respondent curve is more than two standard deviations from the mean of the item curve, it indicates that perhaps the items are inappropriately easy or difficult for the respondents. Respondents found it easiest to say that pain interfered with their work and daily activities. Respondents found it most difficult to say that pain interfered with their social relationships and attending school. Upon visual inspection, it appears that the item difficulty distribution did not mirror the person ability distribution. A significant ceiling effect was noticed where 46% of respondents were at least two standard deviations of measure logits above the mean of the item difficulty distribution. Lastly, a significant content gap was observed for less difficult items: items which respondents find easy to say that pain interferes with an area of life.

### 3.2. Construct Validity

Unidimensionality assessments indicated that 64.2% of the raw variance in the sample could be explained by the measures and the difference between the variance observed in the sample and expected by the model was 0.6%. The unexplained variance in the first contrast had an eigen value of 2.0 and the point-biserial measure correlation for all items were positive and greater than 0.75. Values for each test parameter assessing dimensionality can be found in Table 2.

### 3.3. Scale Function

There were at least 20 responses in each response category. The distribution of response frequencies advanced from one category to the next (“Very much” (*n* = 21); “Quite a bit” (*n* = 38); “Somewhat” (*n* = 113); “A little bit” (*n* = 153); “Not at all” (*n* = 530)). The raw data for response category frequencies are displayed in Table 3.

**Figure 2:**
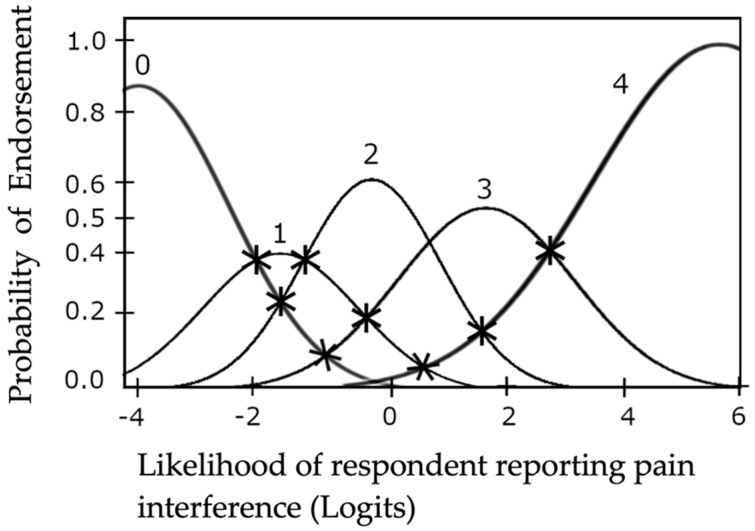
Response Probability Curves for Pain Interference in the lives of participants. * indicates an Andrich threshold. Response options: 0 = “Very much”; 1 = “Quite a bit”; 2 = “Somewhat”; 3 = “A little bit”; 4 = “Not at all”.

The observed average, sample expected responses and Andrich thresholds advanced monotonically from {−1.63 to 3.11}, {−1.89 to 3.11}, and {−2.05 to 2.41} respectively. Outfit mean-squares ranged from {0.72 to 1.35} and infit mean-squares ranged from {0.78 to 1.33}. Coherence of ratings which imply measures and measures which imply ratings for “Quite a bit”, “Somewhat”, “A little bit” and “Not at all” were greater than 40%. “Very much” had coherence of 10% for ratings which imply measures and 33% for measures which imply ratings. Andrich logits were between {3.41 and 3.66}. Item separation was 2.61. Values for each test parameter assessing scale functionality can be found in Table 2. Response probability curves were visually examined and are displayed in Figure 2. To examine the figure, the probability of responding to a specific response option (e.g., “Very much” or “A little bit”, etc.) is measured on the vertical axis from 0.00 to 1.0. The ability of the respondent in logits is measured on the horizontal axis. Each response option will have a response probability peak at a unique ability along the scale. Ideally for an appropriately functioning Guttman Scale, the response option curves will align from easiest, “Very Much”, to most difficult, “Not at all”, along the x axis and be distinct from one another (i.e., minimal overlap). Andrich thresholds are where an individual at that ability level has an equal probability of responding to two different response options. Peak 1 (“Quite a bit”) was minimally distinct from peak 0 (“Very Much”) and peak 2 (“Somewhat”). Peak 3 (“A little bit”) was minimally distinct from peak 2 (“Somewhat”) and peak 4 (“Not at all”). Peaks 0 to 4 advanced monotonically from −4 to 6 along person minus item measure logit scale.

### 3.4. Reliability

The internal consistency was measured by Cronbach’s alpha with a result of 0.92. Using Rasch analysis, item separation was 2.61 and item reliability was 0.87. See Table 2 for additional information.

## 4. Discussion

The primary objective of this study was to assess the reliability and validity parameters of a five-item, five-response option Likert-type scale addressing pain interference in the lives of 172 student pharmacists at the University of Arizona. We assessed reliability and internal consistency using Cronbach’s alpha. We used Rasch analysis methods to establish the extent of construct validity, content validity, and scale functioning. We assumed that no set of items will fully satisfy strict parameters for reliability, validity and scale functioning [18,24]. Therefore, we attempted to determine whether item parameter estimates were robust enough to establish sufficient and comparable reliability and validity to permit relatively unbiased scaling of pain interference among student pharmacists. We did this by comparing estimated parameters to recommended and published values in literature as well as referencing prior validation studies of pain interference items. Our study is unique in that no other validation study has yet been published on a pain interference scale using Rasch analysis to assess scale functioning. Rasch analysis is distinct among IRT analytical techniques in that it assesses the extent to which scaled items function to produce interval level data and behave like a Guttman Scale, a scale characterized by a positive correlation between the ability of respondents and the probability of respondents endorsing more difficult items [18]. For this reason, there are no studies available for direct comparison of the estimated parameters of our specific Rasch analysis tests. However, extensive recent research has been carried out to validate pain interference instruments in various administration formats, populations and settings [14,26,27,28,29]. Results of that research are mentioned, and general comparisons are made wherever appropriate.

Results of the reliability test for internal consistency (Cronbach’s alpha = 0.92) suggest that the Pain Interference Scale satisfies the minimum recommended threshold of 0.9 for high internal consistency as set by the National Quality Forum [24]. The National Institute of Health-Funded Patient-Reported Outcomes Measurement System (PROMIS) sets a Cronbach’s alpha target of greater than 0.85 for pain interference instruments [26]. Wright and Panchapakesan [25] suggested item separation of 2 logits and Linacre [22] suggested ideal ranges for infit and outfit mean-squares and z-standard scores for minimizing inclusion of misfitting items. Item separation and all mean-square and z-standard values were within recommended thresholds. This suggests that the data has sufficient reliability based on published standards.

All unidimensionality tests exceeded recommended thresholds suggested by Linacre [21]. Therefore, the data strongly suggests that the items in the Pain Interference Scale measure the same central construct. Two pharmacist researchers with experience in pain management examined the face validity of the Pain Interference Scale and agreed that the items appeared to measure pain interference.

In accordance with recommendations made by Wright and Masters [20] for assessing content validity, we visually examined the Item-Person Map, Figure 1, to determine whether item difficulty distribution mirrored the person ability distribution. A ceiling effect was observed in the Item-Person Map suggesting that a substantial content gap exists. We examined the most difficult and easy items to endorse. It was most difficult for students to say that pain interfered with social relationships and attending school and least difficult to say that pain interfered with work and daily activities. The content gap existed for items which students would easily endorse that pain interfered in an area of their lives. Therefore, it is recommended that items are added to the Pain Interference Scale to capture student pharmacists’ experiences with pain interference more fully.

It is of great importance to any university program that their students succeed as professionals and as citizens. For these student pharmacists, the most common pain locations were in the head, abdomen, legs and feet [13]. Therefore, we suggest that future research address areas of pain interference in the lives of professional students which include sleep, daily activities, relaxation, concentration, memory, and creativity. Knowing more about how pain affects these students’ engagement in high stakes settings, such as objective structured clinical examinations (OSCE), may be key to understanding the impact of pain on their professional functionality and, perhaps, the societies they serve. Because the 56-item PROMIS-PI instrument and 4-item, 6-item and 8-item v1.0 short forms are commonly used in clinical settings, we suggest several PROMIS-PI items published in the public domain by Health Measures, a service by Northwestern University, from the PROMIS—Ca Item Bank v1.1—Pain Interference questionnaire [14,26,27,29,30]. These items can be found in Table 4.

Scale functioning was assessed using recommendations by Linacre [25] and Wright and Masters [20]. All recommended estimated parameter thresholds for scale functioning were met by the data. Though probability curves, Figure 2, were minimally distinct they were determined to be “sufficiently” distinct for each response category to be considered essential for scale functioning based on Wright and Masters’ recommendations [20]. Lastly, the response options progressed in order of difficulty as would those of a Guttman Scale [20]. Therefore, data strongly suggests that the Pain Interference Scale functions as a Guttman Scale and produces interval level data. It may be appropriate to perform parametric Classical Test Theory analysis on these data.

Our study has several limitations inherent in conducting validation and reliability studies of surveys. This survey was administered to student pharmacists whose course of study includes patient pain management strategies. Some qualities of our dataset may limit the generalizability of our results. Neither validity nor reliability of an instrument are ever established definitively or permanently because validity and reliability of an instrument are bound to the context in which the instrument is used [18]. For example, it would be inappropriate to assume the same extent of construct validity found in this study while using the Pain Interference Scale in a population of pharmacists. Validity is an iterative process where evidence for an instruments’ validity is accumulated across multiple populations, cultures, time periods, etc. However, our study is valuable as it begins to establish validity and reliability evidence for the instrument. Similarly, we suggest that further validation and reliability studies are needed to determine the Pain Interference Scale’s appropriate use in other populations.

## 5. Conclusions

We assessed validity and reliability evidence for the Pain Interference Scale using IRT and CTT tests. We conclude that the Pain Interference Scale appears to demonstrate construct validity in terms of face validity. However, it appears that there are significant content gaps. Therefore, we failed to establish the content validity of the Pain Interference Scale in this population. Recommendations are made including introducing items assessing pain interference in daily activities, concentration, and relaxation. We conclude from our assessment of the scale functioning that the Pain Interference Scale appears to produce ordinal level data which approximates interval level data in this population. Therefore, it may be appropriate to use parametric Classical Test Theory analysis on this dataset. Because our validation study is unique in that it included Rasch analysis of scale functioning and was assessed in a unique subpopulation, it may serve as a model for other validation studies of pain interferences instruments in specialized contexts.

## Figures and Tables

**Table 1:** Pain Interference Scale.

How Much Does Pain Interfere with Your:	Not At All	A Little Bit	Somewhat	Quite A Bit	Very Much
Daily activities?	◯	◯	◯	◯	◯
Ability to participate in leisure activites?	◯	◯	◯	◯	◯
Ability to work?	◯	◯	◯	◯	◯
Ability to attend school?	◯	◯	◯	◯	◯
Relationships with other people?	◯	◯	◯	◯	◯

**Table 2:** Summary of validity, reliability, and scale functioning studies of the Pain Interference Instrument.

Test		Threshold	Pain Interference Items				
			Daily Activities	Leisure Activities	Work	School	Relationships
Reliability	Cronbach’s Alpha	>0.7 [20]	0.92				
	Item Separation and Reliability	Separation/Reliability >2/>0.8 [21]	2.61/0.87				
	Item Infit/Outfit	0.6 < MNSQ < 1.4 −2 < ZSTD < 2 [22]	(1.23/1.16) (1.60/1.16)	(0.94/0.94) (0.37/1.16)	(0.70/0.72) (−2.39/−2.13)	(0.94/0.86) −0.40/−0.87)	(1.30/1.04) (1.85/0.28)
Dimensionality and Item correlation	Raw variance explained by measures	>60% [23]	64.2%				
	The difference between modeled and empirical raw variance explained by measures	≈0% [23]	0.6%				
	Eigen value for the unexplained variance in the 1st contrast	>2 [23]	2.0				
	Point-biserial correlations between x and y	>0.2 and positive [23]	0.84	0.85	0.90	0.83	0.77
Content and Criterion Validity	Item-Person Map	Person ability and item difficulty “mirror” each other; assess for ceiling or floor effects [24]	Ceiling effect (See Figure 1. “Item-Person Map”)				
	Redundancy	Items with the same difficulty level [24]	No redundancy (See Figure 1. “Item-Person Map”)				
	Item difficulty gaps	Z-value <2 [24]	Large high difficulty gap (See Figure 1. “Item-Person Map”)				
			Response Options				
			Very much	Quite a bit	Somewhat	A little bit	Not at all
Scale function	Frequency response category used	≥10 per category and regular increase across categories [22]	21	38	113	153	530
	Observed average and expected	Advances monotonically [22]	−1.63	−1.10	0.46	2.01	3.11
	Andrich threshold	Moves from − to 0 to + [22]	REF	−2.05	−1.33	0.97	2.41
	Coherence with rating which imply measures and measures which imply ratings	Measures imply ratings/ratings imply measures >40% per category [22]	(33%/10%)	(47%/58%)	(63%/49%)	(49%/68%)	(77%/65%)
	Anchor MNSQ	(Infit/Outfit) Anchors (“Very much” and “Not at all”) have largest MNSQ [22]	(1.33/1.35)	(0.93/0.94)	(0.78/0.72)	(0.97/0.87)	(1.20/1.13)
	Andrich measures	|1.4| < *X* < |5| [22]	−3.41	−1.75	−0.13	1.74	3.66
	Probability curves	Advancing, distinct and well separated peaks [24]	Response option 1 is not distinct from 0 or 2; response option 3 is minimally distinct from 2 and 4; advances sequentially (see Figure 2) “Probability Curves”				

Key: Inlier-pattern-sensitive fit statistic (INFIT); outlier-sensitive fit statistic (OUFFIT); mean-square (MNSQ); z-standardized (ZSTD).

**Table 3:** Response option frequency and percent of total responses to Pain Interferences Scale items.

How Much Does Pain Interfere with…?	Very Much *n* (%)	Quite A Bit *n* (%)	Somewhat *n* (%)	A Little Bit *n* (%)	Not At All *n* (%)
Daily activities (*n* = 171)	1 (1)	9 (5)	32 (19)	40 (23)	89 (52)
Leisure activities (*n* = 171)	5 (3)	7 (4)	22 (13)	34 (20)	103 (60)
Work (*n* = 170)	4 (2)	11 (6)	25 (15)	37 (22)	93 (55)
School (*n* = 171)	5 (3)	6 (4)	19 (11)	24 (14)	117 (68)
Relationships (*n* = 172)	6 (3)	5 (3)	15 (9)	18 (10)	128 (74)

**Table 4:** Suggested items that can be added to fill content gaps.

Item #	PROMIS Bank v1.1- Pain Interference Item ^1^
Painin8	How much did pain interfere with your ability to concentrate?
Painin22	How much did pain interfere with work around the home?
Painin14	How much did pain interfere with doing your tasks away from home (e.g., getting groceries, running errands)?
Painin19	How much did pain make it difficult to fall asleep?

^1^ All suggested items are measured on a 5-point scale with response options ranging from 0 = “Not at all” to 4 = “Very much”.

## Data Availability

The data presented in this study are available on request from the corresponding author.
